# Spontaneous *asj-2J* Mutant Mouse as a Model for Generalized Arterial Calcification of Infancy: A Large Deletion/Insertion Mutation in the *Enpp1* Gene

**DOI:** 10.1371/journal.pone.0113542

**Published:** 2014-12-05

**Authors:** Qiaoli Li, C. Herbert Pratt, Louise A. Dionne, Heather Fairfield, Son Yong Karst, John P. Sundberg, Jouni Uitto

**Affiliations:** 1 Department of Dermatology and Cutaneous Biology, Sidney Kimmel Medical College at Thomas Jefferson University, Philadelphia, Pennsylvania, United States of America; 2 The Jackson Laboratory, Bar Harbor, Maine, United States of America; Los Angeles, United States of America

## Abstract

Generalized arterial calcification of infancy (GACI), an autosomal recessive disorder caused by mutations in the *ENPP1* gene, manifests with extensive mineralization of the cardiovascular system. The affected individuals in most cases die within the first year of life, and there is currently no effective treatment for this disorder. In this study, we characterized a spontaneous mutant mouse, *asj-2J*, as a model for GACI. These mice were identified as part of a phenotypic deviant search in a large-scale production colony of BALB/cJ mice at The Jackson Laboratory. They demonstrated a characteristic gait due to stiffening of the joints, with phenotypic similarity to a previously characterized *asj* (“ages with stiffened joints”) mouse, caused by a missense mutation in the *Enpp1* gene. Complementation testing indicated that *asj-2J* and *asj* were allelic. PCR-based mutation detection strategy revealed in *asj-2J* mice a large, 40,035 bp, deletion spanning from intron 1 to the 3′-untranslated region of the *Enpp1* gene, coupled with a 74 bp insertion. This was accompanied with a significant reduction in the plasma PP_i_ concentration and reduced PP_i_/P_i_ ratio. As a consequence, extensive aberrant mineralization affecting the arterial vasculature, a number of internal organs, and the dermal sheath of vibrissae, a progressive biomarker of the ectopic mineralization process, was demonstrated by a combination of micro computed tomography, histopathology with calcium-specific stains, and direct chemical assay of calcium. Comparison of the *asj* and *asj-2J* mice demonstrated that the latter ones, particularly when placed on an acceleration diet high in phosphate and low in magnesium, had more extensive mineralization. Thus, the *asj-2J* mouse serves as a novel model for GACI, a currently intractable disorder.

## Introduction

Generalized arterial calcification of infancy (GACI) is a severe ectopic mineralization disorder affecting primarily the arterial blood vessels in humans. The disease is often diagnosed by prenatal ultrasound, and the affected individuals in most cases die within the first year of life from cardiovascular complications [1], [2]. GACI is inherited in an autosomal recessive fashion, and most cases are due to mutations in the *ENPP1* gene, which encodes ectonucleotide pyrophosphatase/phosphodiesterase 1 (ENPP1), an enzyme that hydrolyses ATP to AMP and inorganic pyrophosphate (PP_i_) [3]. Under physiological conditions, PP_i_ serves as a powerful anti-mineralization factor, and with reduced ENPP1 activity in GACI, the ratio of inorganic phosphate (P_i_) to PP_i_ increases creating a pro-mineralization environment and allowing ectopic tissue mineralization to ensue [4]. There is currently no effective treatment for GACI.

A number of mouse models recapitulating the clinical features of human diseases with vascular mineralization have been described [5], [6]. One of them, the *asj* mouse, was recently identified as a result of ENU treatment in The Jackson Laboratory Neuromutagenesis Program [7]. These mice were originally noted to demonstrate a stiff posture, abnormalities in the front legs, and a progressive, age-associated stiffening of the joints. Pathological examination at seven months of age revealed very stiff and unbendable joints with severe osteoarthritis and mineralization, and consequently, this mutant mouse was designated as “ages with stiffened joints (*asj*)”. Molecular characterization of these mice identified a missense mutation (p.V246D) in the *Enpp1* gene which resulted in markedly reduced ENPP1 enzymatic activity, ∼24% of the level in control mice, accompanied with a marked, >80% reduction in plasma PP_i_ concentration [7]. Necropsy of the homozygous *asj* mice revealed extensive mineralization affecting dermal sheath of vibrissae, an observation we have previously made in *Abcc6^tm1Jfk^* knockout mice (designated hereafter as the *Abcc6^−/−^* mouse), a model for another ectopic mineralization disorder, pseudoxanthoma elasticum (PXE) [8]. The *asj* mouse also demonstrated extensive vascular mineralization, particularly when placed on a so-called “acceleration diet”, enriched in phosphate and low in magnesium content. We have also shown that this diet markedly enhances the vascular mineralization in *Abcc6^−/−^* mice [9], [10].

More recently, a spontaneous mouse phenotype with similarity to that of *asj* mouse was noted as part of the phenotypic deviant search in The Jackson Laboratory BALB/cJ large-scale production colony. In this study, we have characterized the phenotypic and histopathologic features of this spontaneous mutant mouse, designated as *asj-2J*, and we have identified a large deletion/insertion mutation in the *Enpp1* gene.

## Materials and Methods

### Mouse maintenance and diets

**Figure 1:**
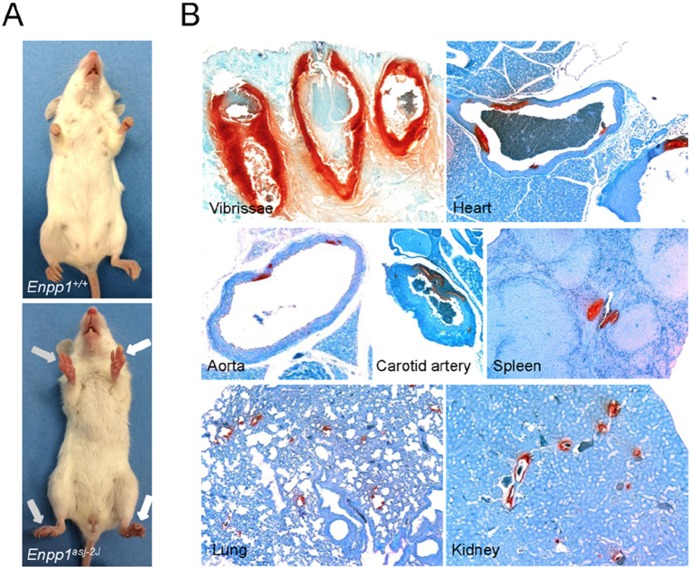
Phenotypic presentation and extensive ectopic mineralization of *asj-2J* mice. A: Note the appearance of stiffened front and hind feet in *asj-2J* mice (arrows, lower panel) in comparison to a wild-type littermate. B: Note the extensive mineralization (red color) in the dermal sheath of vibrissae, arterial blood vessels, and various internal organs in *asj-2J* mice, as visualized by special stain (Alizarin Red).

The spontaneous mutant mouse (*asj-2J*) was discovered in 2011 at The Jackson Laboratory (JAX; Bar Harbor, ME) in a large-scale production colony of BALB/cJ mice. Two female mutant mice were initially identified by their slow, hobbling gate due to stiffened joints that worsens as they age, as well as their overall sickly appearance (Figure 1). The deviant mice were transferred to the Mouse Mutant Resource (MMR) at JAX for further study. Upon arrival in the MMR, ovarian transplants were performed on the two affected mice to facilitate breeding. Subsequently, mating of the ovarian transplant recipients to a BALB/cJ wild-type male, followed by random intercrossing of F1 mice revealed an autosomal recessive mode of inheritance. These procedures were approved by The Jackson Laboratory’s Institutional Animal Care and Use Committee and performed in accordance with National Institutes of Health guidelines for the care and use of animals in research.

The *asj-2J* mice were transferred to the Animal Facility of Thomas Jefferson University where they were maintained in a climate-controlled environment. *Enpp1* wild type mice as well as heterozygous and homozygous *asj-2J* mutant mice were generated from heterozygous matings. Mice were maintained either on standard laboratory diet (Laboratory Autoclavable Rodent Diet 5010; PMI Nutritional International) or fed an ‘acceleration diet’ (Harlan Teklad, Rodent diet TD.00442, Madison, WI), which we have previously shown to accelerate the ectopic mineralization in *Abcc6^−/−^* mice [9], [10]; this diet is enriched in phosphorus and has reduced magnesium content. The mice were euthanized by CO_2_ asphyxiation with the use of methods approved by the American Veterinary Medical Association and subjected to necropsy, as described previously in detail [11]. Mouse handling and care were followed according to animal welfare policies of the U.S. Public Health Service. All protocols were approved by the Institutional Animal Care and Use Committee of Thomas Jefferson University.

### Genotyping and gene sequencing

**Figure 2:**
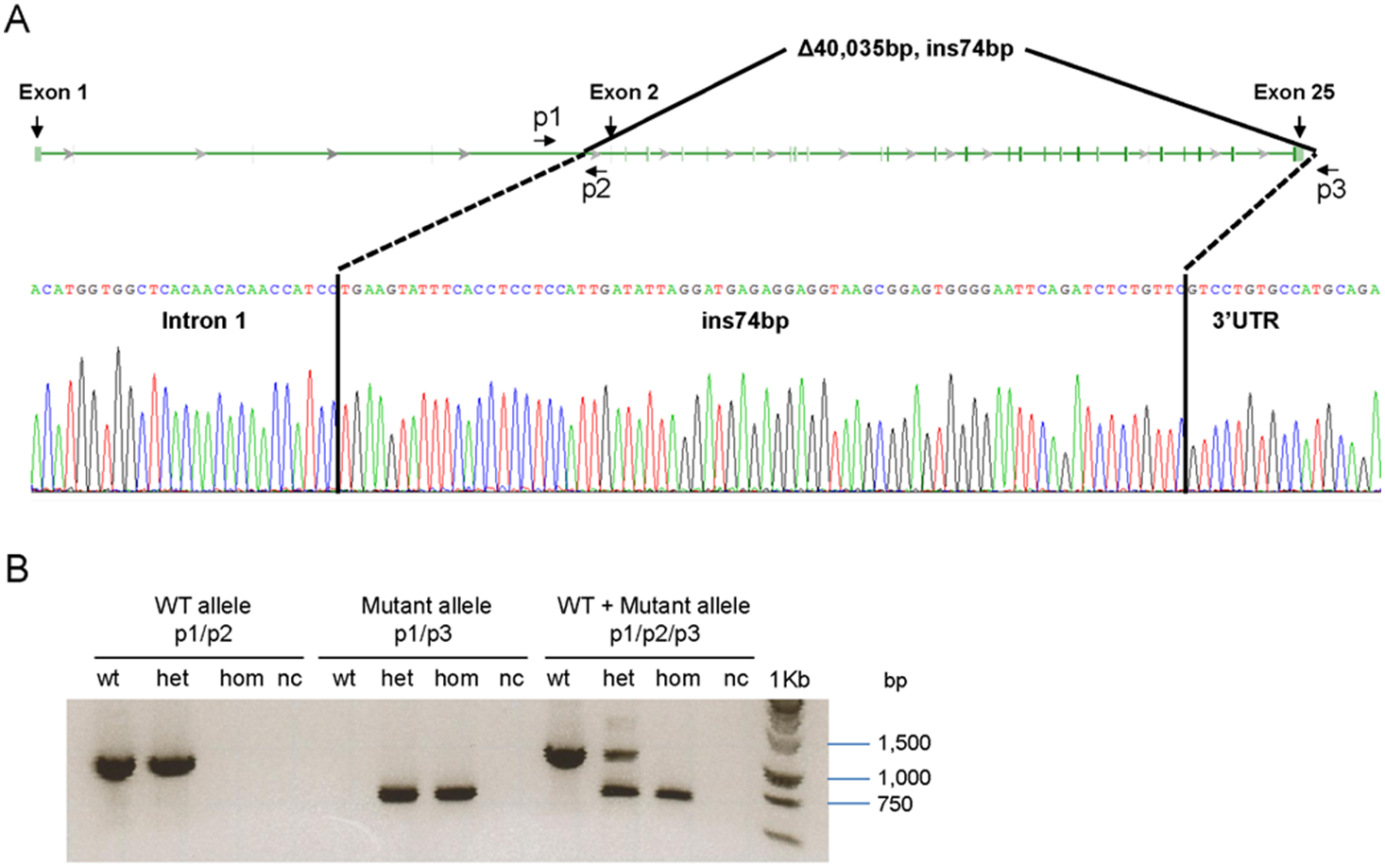
Schematic presentation of the large deletion/insertion mutation in the *Enpp1* gene in *asj-2J* mice, and PCR-based genotyping. A: Note the 40,035 bp deletion extending from intron 1 to the 3′-untranslated region of the gene. The deleted segment is replaced by insertion of a 74 bp fragment which is derived from the 3′ UTR of the gene. B: Development of specific primers (p1/p2/p3), with positions shown in A, allowed genotyping of the wild-type (WT) and mutant alleles and identification of wild-type, heterozygous and mutant homozygous mice. nc: negative control without DNA.

Genomic DNA was isolated in tail clips from mice using DNeasy Blood & Tissue kit (Qiagen Inc., Valencia, CA). The entire coding region consisting of 25 exons and intron/exon boundaries of *Enpp1* sequences were amplified using PCR primers (available upon request). The *asj-2J* mice were found to have a large deletion of ∼40 kb extending from intron 1 up to 3′UTR of the *Enpp1* gene, with a 74 bp insertion (see Results). Three primers were designed for amplification of the wild-type, mutant, or both alleles in the same reaction. The primers were the following: p1, 5′-TCAGTGATTGGTCAACAGACACCT-3′ (in intron 1); p2, 5′-GGAAGACATGAATAGCAACTACCTG-3′ (in intron 1); and p3, 5′-CTTTGGTTATTGGAGGAGACAGAAA-3′ (in 3′UTR); for their positions along the *Enpp1* gene, see Fig. 2. The primer pair p1/p2 produced a 1,042-bp wild-type allele, while primers p1/p3 resulted in a 654-bp mutant allele. Gene sequencing was performed at the Kimmel Cancer Center Nucleic Acid Facility at Thomas Jefferson University using the BigDye Terminator v.3.1 Cycle Sequencing Kit (Applied Biosystems, Foster City, CA). Products were analyzed on the 3730 DNA Analyzer (Applied Biosystems) and the results were visualized with Chromas software (Technelysium, South Brisbane, Australia). Repetitive sequences were analyzed by RepeatMasker program (http://www.repeatmasker.org/cgi-bin/WEBRepat Masker).

### Small-animal computed tomography (micro CT scan)

*Enpp1* wild-type and *asj-2J* mice were examined for mineralization at 12 weeks of age by CT, as described previously [7], [12]. Briefly, mice were anesthetized with a xylazine-ketamineacetopromazine cocktail (160 µl per 25 g body weight of 10 mg/kg xylazine, 200 mg/kg ketamine, 2 mg/kg acetopromazine) and then scanned with a MicroCAT II (ImTek Inc., Oak Ridge, TN). A 3-dimensional facial rendering was created for each mouse using Amira software, version 3.1 (Visualization Sciences Group, Burlington, MA).

### Histopathological analysis

Muzzle skin (left side) and internal organs from euthanized mice were fixed in 10% phosphate-buffered formalin, routinely processed, and embedded in paraffin. Tissues were sectioned (6 µm) and stained with hematoxylin and eosin (H&E) and Alizarin red using standard procedures. Slides were examined under light microscopy for tissue mineralization [13].

### Quantification of calcium and phosphate

To quantify the calcium deposition in various mouse tissues, muzzle skin (right side), abdominal aorta, right carotid artery and right kidney were harvested and decalcified with 0.15 N HCl for 48 hours (skin, aorta and carotid artery) or with 0.6 N HCl for 1 week (kidney) at room temperature. The calcium content in these samples as well as in serum was determined colorimetrically by the *o*-cresolphthalein complexone method [Calcium (CPC) Liquicolor; Stanbio Laboratory, Boerne, TX]. The values for calcium were normalized to tissue weight and in case of carotid artery to its total length. Serum calcium concentrations were measured using the same assay. The phosphate concentration of serum was determined with Malachite Green Phosphate Assay kit (BioAssay Systems, Hayward, CA).

### Plasma collection and inorganic pyrophosphate assay

Whole blood was collected by cardiac puncture into heparin coated blood collection tubes and kept on ice until separation of plasma and erythrocytes by centrifugation. The plasma was collected, depleted of platelets by filtration (2,200×g at 4°C for 20 min) through a Centrisart I 300-kDa mass cutoff filter (Sartorius, New York, NY, USA), and stored at −80°C until further processing. PP_i_ in plasma was measured by an enzymatic assay using uridine-diphosphoglucose (UDPG) pyrophosphorylase, with modifications, as described previously [4], [14], [15].

### Statistical analysis

The comparisons in different groups of mice were completed using two-sided Kruskal-Wallis nonparametric tests which is comparable to one-way analysis of variance, but without the parametric assumptions. Fisher’s exact test was used to determine the difference between proportions in mineralization phenotypes in mice. Chi-squared test was used to compare observed data with expected data according to Mendelian inheritance. All statistical computations were completed using SPSS version 15.0 software.

## Results

### Characterization of *asj-2J* mice

The *asj-2J* mutant mice were identified at The Jackson Laboratory as part of the phenotypic deviant search in a large-scale production colony of BALB/cJ mice, as described in Materials and Methods. Putative homozygous mice, when maintained on standard mouse diet, looked hunched at 8 weeks, and the whole body appeared stiffened at about 12 weeks of age. In particular, stiffening of the front paws was noted, similar to previously published *asj* mice [7] (Fig. 1A). The distribution of the mutant genotype and the gender of the pups were subsequently determined in 86 mice representing a total of 19 litters as a result of heterozygous matings. The distribution of the genotype for the homozygous wild-type, heterozygous, and homozygous mutant mice, 23∶37∶26, did not statistically differ from the expected 1∶2∶1 ratio (Chi-squared test; p>0.01), and the male/female ratio, 41∶45, was approximately 1∶1, all consistent with autosomal recessive inheritance without gender preference.

Complementation testing was undertaken using the previously identified mutant mouse strain, C57BL/6J-*Enpp1^asj^*/GrsrJ, designated as the *asj* mouse, with similar phenotype. Heterozygous *asj* females were mated to a heterozygous male *asj-2J* mouse resulting in one affected pup in the first litter of six pups, thus *asj-2J* was suggested to be a re-mutation in the *Enpp1* gene. The strain was officially named BALB/cJ-*Enpp1^asj-2J^*/GrsrJ.

### Identification of a deletion/insertion mutation in *Enpp1*

Based on phenotypic similarities and the complementation tests between the *asj* and *asj-2J* mice, it was hypothesized that these mice might be allelic and harbor mutations in the *Enpp1* gene. To search for mutations in the *Enpp1* gene, primer pairs were developed for amplification of all 25 exons of the *Enpp1* gene, together with flanking intronic sequences. PCR amplification of exon 1, but none of the remaining exons 2–25, resulted in a PCR product in homozygous mice, whereas products with correct *Enpp1* sequences were obtained in heterozygous and wild-type mice. This result suggested the presence of a large deletion spanning most of the *Enpp1* gene. In attempts to identify the break point downstream from the *Enpp1* gene, the next gene, ∼44 kb downstream from *Enpp1*, was identified as *Ctgf* in reverse strand, and the last exon, no. 5, of this gene was subjected to PCR amplification. This approach clearly demonstrated the presence of *Ctgf* in homozygous *asj-2J* mice, indicating that the breakpoint of the large deletion resides somewhere between the end of exon 25 of *Enpp1* and upstream from the end of *Ctgf*. To precisely map the boarders of the deletion, primer pairs at 5 kb interval were designed to span this critical region and PCR amplifications were performed. This approach mapped the 5′ end of the deletion within intron 1 at 2,376 bp upstream of exon 2 of *Enpp1* and the 3′ end of the deletion at nucleotide position 199 downstream from the stop codon (TGA) in exon 25 of *Enpp1* within the 3′-untranslated region of the gene (Fig. 2A). To verify the precise breakpoints of the deletion, primer pairs within intron 1 of *Enpp1* (forward primer) and downstream from the identified breakpoint at the 3′ end (reverse primer) were generated. Sequencing of this region indicated that the mutation consisted of a large deletion of 40,035 bp, but also harbored an insertion of a 74 bp DNA fragment (Fig. 2A). A 74 bp segment with identical sequence was identified in the 3′-untranslated region of the *Enpp1* gene in a region of mouse chromosome 10qA4 as part of the Long Interspersed Nuclear Element (LINE) L1MDa.

Analysis of the sequences spanning the deletion breakpoints was performed to detect possible repetitive elements using the RepeatMasker program. The breakpoint in intron 1 of the *Enpp1* gene was adjacent to ∼5 kb Long Terminal Repeat (LTR) with a high content of repetitive elements (99.3%). The breakpoint at 3′-untranslated region of the *Enpp1* gene was adjacent to a ∼500 bp LINE, specifically L1MDa element (70.1% repetitive elements), where the 74 bp insertion resides. These repeats may have played a role in mediating the large deletion in the *Enpp1* gene in the *asj-2J* mice.

Precise knowledge of the breakpoints in the mutant mouse, in comparison to the wild-type sequence, allowed us to develop a PCR based genotyping strategy utilizing a common forward primer and separate reverse primers for the mutant and the wild-type allele. In the case of wild-type allele, a 1,042 bp PCR product results, while mutant allele yields a 654 bp band (Fig. 2B). This rapid genotyping allows us to routinely identify homozygous mutant, heterozygous and wild-type littermates in heterozygous crossings.

### Demonstration of aberrant mineralization in *asj-2J* mice

**Figure 3:**
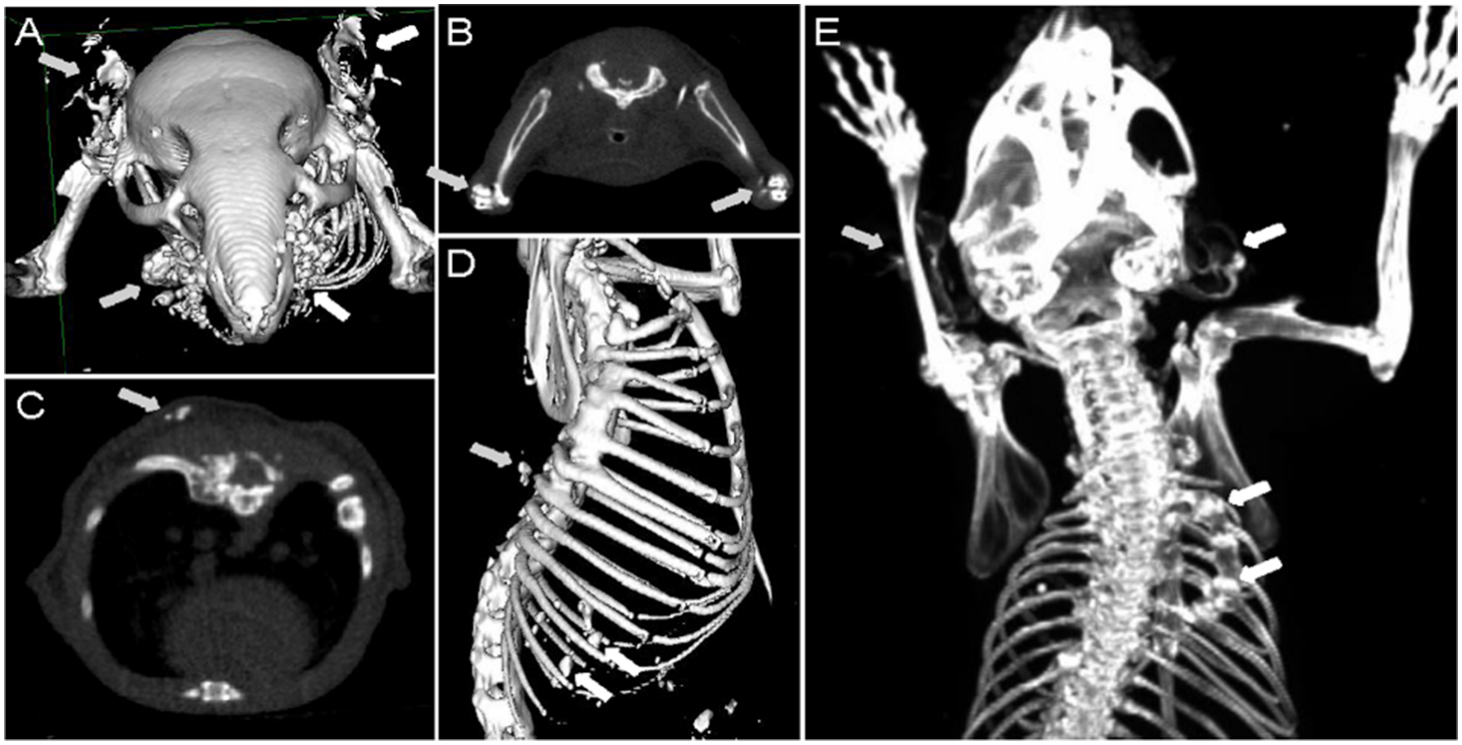
Non-invasive demonstration of ectopic mineralization in *asj-2J* mice by micro CT. Note the extensive mineralization in the muzzle skin containing the dermal sheath of vibrissae and in the ears (A, arrows); in the juxta-articular connective tissue in the elbows (B, arrows); axial view of dorsal artery (C, arrow); spinal and intercostal tissues (D, arrows); and in the right rib fusion leading to scoliosis as well as in the ears (E, arrows).

Considering the notion that *asj-2J* mice are allelic to *asj* mice, which demonstrate considerable mineralization of soft connective tissues, the *asj-2J* mice were examined for mineralization in the muzzle skin containing the dermal sheath of vibrissae, the first site of mineralization in *asj* as well as *Abcc6^−/−^* mice [7], [8]. This was first done non-invasively in 3 month old mice by micro CT analysis, and the results demonstrated extensive mineralization corresponding to the muzzle skin as well as in juxta-articular connective tissues, spinal and intercostal tissues, and in various arteries (Fig. 3). The mineralization of the dermal sheath of vibrissae was subsequently confirmed by histopathologic examination of the muzzle skin. Histopathology of skin sections with Alizarin red stain demonstrated extensive calcification in *asj-2J* mice at 3 months of age when kept on control diet (Fig. 1B). Complete necropsies of the mice also revealed extensive arterial mineralization in descending thoracic aorta, carotid artery, and in a number of internal organs, including the heart, spleen, lung, and the kidney (Fig. 1B).

**Figure 4:**
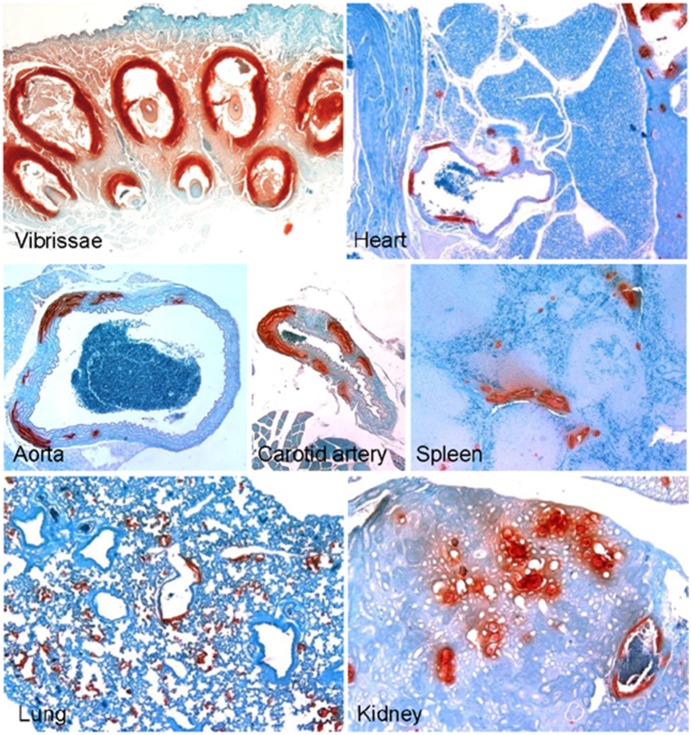
Enhanced mineralization in *asj-2J* mice placed on “acceleration diet”, rich in phosphate and low in magnesium. The mice were placed on this diet at 4 weeks of age and necropsy was performed at 12 weeks. Note extensive mineralization as visualized by special stain (Alizarin Red). Assessment of the histopathology suggested that the mineralization was more extensive in mice kept on acceleration diet compared to the same mice kept on control diet (compare mineralization in Fig. 1B).

**Figure 5:**
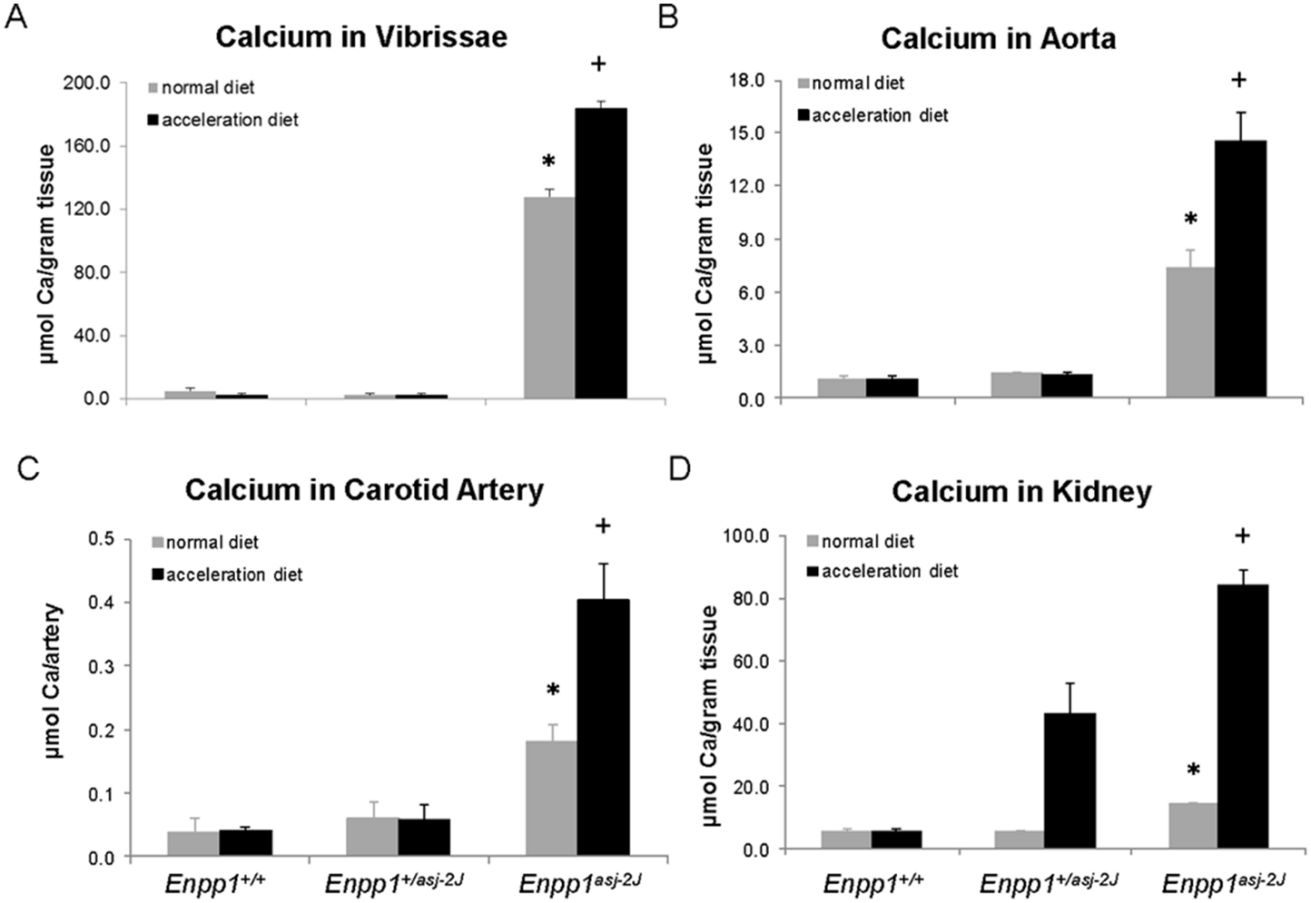
Quantitation of ectopic mineralization by chemical assay of calcium content in the muzzle skin (A), aorta (B), carotid artery (C), and kidney (D) in 12-week old *asj-2J* mice kept either on normal mouse diet or placed on acceleration diet at 4 weeks of age. The statistical analysis was performed with Kruskal-Wallis nonparametric test; *, p<0.01 in comparison to wild-type *Enpp1^+/+^* mice; ^+^p<0.01 as compared with *asj-2J* mice kept on regular diet. Values are mean ± SE, n = 6–12 per group.

We have previously demonstrated that a special diet, so-called “acceleration diet”, low in magnesium and high in phosphorus, facilitates the mineralization process in *asj* and *Abcc6^−/−^* mice [9], [10]. In this study, the *asj-2J* mice were placed on the acceleration diet at 4 weeks of age, at the time of weaning, and they were necropsied at 12 weeks of age and analyzed for mineralization and compared to that in the same mice kept on control diet. Histopathologic examination of the muzzle skin indicated extensive mineralization that appeared semi-quantitatively to be more abundant in *asj-2J* mice on the acceleration diet than in mice on the control diet (compare Fig. 1 and Fig. 4). To quantitate the amount of mineralization, a piece of muzzle skin, the abdominal aorta, carotid artery, and kidney were dissected and calcium deposits were solubilized, after which the calcium content was measured by a colorimetric chemical assay. The results indicated that tissue mineralization in homozygous mice placed on acceleration diet was higher than in the corresponding mice kept on control diet (Fig. 5 and Table 1). It should be noted that the heterozygous *Enpp1^+/asj-2J^* mice when on control diet did not show any evidence of aberrant tissue mineralization; however, the same mice, when placed on acceleration diet, demonstrated increased mineralization in the kidney (Table 1 and Fig. 5). This observation may reflect reduced capacity of the kidney to respond to the diet rich in phosphate, as noted in our previous studies [16]. Finally, the calcium contents in these tissues in males (n = 4) and females (n = 8), when analysed separately, were not statistically different (Kruskal-Wallis test; p = 0.9).

**Table 1:** Aberrant tissue mineralization in *Enpp1^+/+^*, *Enpp1^+/asj-2J^* and *Enpp1^asj-2J^* mice on normal and acceleration diet compared with *Enpp1^asj^* mice on acceleration diet.

Mouse	Number ofmice examined	Soft tissue mineralization (%)								
		Vibrissae	Liver	Kidney	Heart	Aorta	Eyes	Carotidartery	Lung	Spleen
Normal diet:										
* Enpp1^+/+^*	8	0	0	0	0	0	0	0	0	0
* Enpp1^+/asj-2J^*	11	0	0	0	0	0	0	0	0	0
* Enpp1^asj-2J^*	12	100	8	100	100	67	33	83	92	67
Acceleration diet:										
* Enpp1^+/+^*	8	0	0	88	0	0	0	0	0	0
* Enpp1^+/asj-2J^*	8	0	0	100	0	0	0	0	0	0
* Enpp1^asj-2J^*	6	100	100**	100	100	100	100*	100	100	100
Acceleration diet:										
* Enpp1^asj^*	9	100	56	100	67	56	44^+^	78	N/A	N/A

Mice were placed on either normal diet or acceleration diet at 4 weeks of age, and tissues were collected for histopathology at 3 months of age. The values represent the number of mice, as percent of all animals examined, affected by mineralization as examined by hematoxylin and eosin stain on one section. Statistical analyses were performed with Fisher’s Exact test. Comparison to *Enpp1^asj-2J^* mice on normal diet: *P<0.05; **P<0.01. Comparison to *Enpp1^asj-2J^* mice on acceleration diet: ^+^ *P*<0.05.

### Comparison of mineralization in *asj* and *asj-2J* mice

As indicated above, C57BL/6J-*Enpp1^asj^* and BALB/cJ-*Enpp1^asj-2J^* mice are allelic in *Enpp1* gene with similar phenotypic features. The mutation in *asj* mice has previously shown to be a missense mutation, p.V246D [7], while the *asj-2J* mice have a large deletion eliminating 24 out of 25 exons, *i.e.*, 57% of the entire gene and 93% of the coding region. To examine whether the differences in the mutations might lead to different degrees of ectopic mineralization, these two mutant mouse strains were placed on acceleration diet at 4 weeks of age, and complete necropsies were performed at 12 weeks, *i.e.*, after 8 weeks on special diet. Examination of the internal organs, including arterial blood vessels, demonstrated considerable mineralization in both mouse strains - however, the mineralization was more frequent and more extensive in *asj-2J* mice as compared to that in *asj* mice (Table 1). The degree of mineralization was also quantitated by chemical assay of calcium in muzzle skin in *asj* and *asj-2J* mice when placed on acceleration diet. The *asj-2J* mice showed significantly higher level of calcium than that in *asj* mice; *asj*: 126.3±5.8 (n = 9) and *asj-2J*: 176.8±4.8 (n = 6) µmol Ca/g tissue (p<0.01).

### Chemical assays in plasma/serum

The *asj-2J* mice were further analyzed by determining the calcium, phosphorus, and inorganic pyrophosphate concentrations in serum/plasma when kept on regular control diet. The results indicated normal levels of serum calcium and phosphate, and consequently the Ca/P ratio was not altered. However, there was a significant reduction, ∼80% in the inorganic pyrophosphate levels in the plasma of *asj-2J* mice in comparison to the wild-type littermates (Table 2). As a consequence, the PP_i_/P_i_ ratio was significantly reduced from 4.2 to 0.88 (×1,000) (p<0.01).

**Table 2:** Calcium, phosphorus and pyrophosphate concentrations in serum/plasma of mice maintained on a regular rodent diet.

Parameter	Concentration (mean ± S.E.)		
	*Enpp1^+/+^* (n = 8)	*Enpp1^+/asj-2J^* (n = 11)	*Enpp1^asj-2J^* (n = 12)
Calcium (mg/dL)	9.38±0.18	9.52±0.15	9.41±0.17
Phosphorus (mg/dL)	9.32±0.32	9.30±0.31	9.37±0.24
Ca/P ratio	1.01±0.03	1.03±0.03	1.01±0.03
PP_i_ (µM)	2.53±0.44	1.78±0.23	0.52±0.08*
PP_i_/P_i_ ratio (×1,000)	4.22±0.74	2.96±0.38	0.88±0.12*

Blood samples were collected by cardiac puncture, Ca and P concentrations were determined in serum, and PP_i_ levels were measured in heparinized plasma after removal of platelets. Statistical significance in comparison to *Enpp1^+/+^* mice: *P<0.01.

## Discussion

GACI is an autosomal recessive disorder that manifests with profound arterial mineralization often diagnosed by prenatal ultrasound. The affected children are born with cardiovascular complications and the majority of them die within the first year of life [1], [2]. GACI in most cases is caused by mutations in the *ENPP1* gene encoding an enzyme, ENPP1, which catalyzes the hydrolysis of ATP to form AMP and PP_i_. The extracellular PP_i_ is a powerful anti-mineralization factor while P_i_ acts as a pro-mineralization factor, and consequently, a physiological PP_i_/P_i_ ratio is critical in preventing ectopic mineralization under normal homeostasis [4]. There are a number of enzymes and transport systems that contribute to the extracellular PP_i_/P_i_ ratio. In addition to ENPP1, these include CD73, a plasma membrane linked enzyme that breaks down AMP to adenosine and P_i_, and tissue non-specific alkaline phosphatase (TNAP) that converts PP_i_ to P_i_ [5], [17]. The activity of TNAP is inhibited by adenosine, and reduced CD73 activity results in reduction of adenosine concentration, thus allowing accelerated conversion of PP_i_ to P_i_ by TNAP, and therefore promotes ectopic mineralization [18]. In addition, a number of transmembrane protein systems have been shown to facilitate transport of PP_i_ and P_i_ from intracellular milieu to the extracellular space, including ankylosis protein (ANK), and type I–III sodium-dependent P_i_ co-transporters, SLC17A1, SLC34, and SLC20 [19], [20], [21]. Finally, recent studies have suggested that ABCC6, a transmembrane transporter, which is nonfunctional in patients with PXE, is required for release of ATP to the extracellular milieu [22]. Thus, in the absence of functional ABCC6, the extracellular concentration of ATP is reduced, and consequently, there is less ATP to serve as a substrate for ENPP1 to generate extracellular PP_i_. Collectively, under normal physiologic conditions, there is a complex pro-mineralization/anti-mineralization network that is required to maintain the normal homeostatic ratio of PP_i_/P_i_ [2], [5]. Mutations in many of the genes controlling this ratio have been shown to result in ectopic mineralization of the soft connective tissues, particularly in the skin and the arterial blood vessels. For example, mutations in the *ENPP1* gene result in GACI, mutations in the *ABCC6* gene underlie PXE, and patients with mutations in the *NT5E* gene, which encodes CD73, develop arterial calcification due to CD73 deficiency (ACDC) [3], [23], [24].

A number of animal models, particularly targeted and spontaneous mutant mice, have been extremely helpful in providing pathomechanistic information on ectopic mineralization in human diseases [5], [6]. In this study, we describe a novel mutant mouse, *asj-2J*, which was identified in the colony breeding program of The Jackson Laboratory. This mouse was noted to have extensive mineralization of the dermal sheath of vibrissae as well as arterial blood vessels, and the mice developed a phenotypic gait due to periarticular mineral deposits. The mineralization phenotype could be significantly accelerated by placing the mice on “acceleration diet”, enriched in phosphate and low in magnesium. The phenotypic similarity of these mutant mice with a previously described *asj* mouse prompted us to test the hypothesis that *asj-2J* mice were allelic, and complementation studies supported the notion that both mice had mutations in the same gene, *Enpp1*. Previous studies have demonstrated that *asj* mice harbor a homozygous missense mutation, p.V246D, reflecting T-to-A transversion in *Enpp1* as a result of ENU treatment [7]. The mutation in *asj-2J* mice was determined to consist of a large deletion spanning from intron 1 all the way to the end of *Enpp1*, thus completely eliminating functional gene in these mice. As a consequence, these mice were shown to have markedly reduced PP_i_ plasma concentrations leading to reduced PP_i_/P_i_ ratio which allows ectopic mineralization to ensue. It should be noted that the *asj-2J* mice developed more extensive mineralization than *asj* mice when placed on the acceleration diet. This may reflect the differences in the type of mutations, the *asj-2J* having a large deletion, essentially comparable to a complete ablation of the gene, while *asj* mice have a missense mutation allowing residual level of ENPP1 activity [5]. In fact, measurements of ENPP1 in the liver of *asj* mice revealed low levels of activity as compared to *Enpp1* wild-type mice, yet there was clearly measurable activity above the background [5]. It should be noted, however, that these two mice were on different inbred strain backgrounds, *asj* on C57BL/6J and *asj-2J* on BALB/cJ, respectively. We have previously demonstrated that different strain backgrounds can influence the degree of mineralization [10], [13], an observation that may impact on our comparison of differences in *asj* and *asj-2J* mice. Nevertheless, since the mutations in the *ENPP1* gene in patients with GACI result in loss-of-function, the *asj-2J* mouse with complete ablation of the gene appears to be a suitable model system to study this disease [3], [25].

In addition to *asj* and *asj-2J* mice, four other mutations in the mouse *Enpp1* gene have previously been described. One of them, so called “tip toe walking” *(Enpp1^ttw/ttw^)* mouse, has been shown to harbor a stop codon mutation, p.G568X, in *Enpp1* [26], while a mouse with similar phenotype *(Enpp1^ttw-Ham^)* was shown to harbor a splice-site mutation c.259+1G>T in *Enpp1* [27]. A genetically engineered knock-out mouse, *Enpp1^tm1Gdg^,* was shown to exhibit phenotypic similarities to *Enpp1^ttw/ttw^* mice [28], while another mutant mouse, *Enpp1^m1Amgn^*, with a p.C397S mutation showed reduced long bone mineral density and crystal-related arthropathy [29]. While the previously published *Enpp1* mutant mice were shown to develop ectopic mineralization, their characterization was largely focused on skeletal alterations and perispinal soft tissue mineralization. Also, while arterial calcification was noted in some of these previously described mice, the histologic lesions were not evident until ∼16–22 weeks of age. In *asj-2J* mice, when kept on either standard mouse diet or the “acceleration diet”, mineralization of the dermal sheath of vibrissae was noted at ∼4 weeks of age. This was accompanied with early demise of *asj-2J* mice, frequently at the age of 12–16 weeks of life. This is likely due to cardiovascular involvement, particular occlusion of small and medium sized arteries. Thus, this mouse displays features of GACI, and can serve as a platform to test various anti-mineralization compounds that could be used for treatment of ectopic mineralization disorders. It is also of interest that a novel *in vivo* model mimicking features of GACI, a zebrafish *enpp1* mutant *dragonfish*, has recently been described [30]. This model was also treated with bisphosphonate etidronate which rescued aspects of the mineralization phenotype in this fish.

A limited number of studies have been published testing the efficacy of bisphosphonates, stable non-hydrolyzable pyrophosphate analogues of pyrophosphate, to counteract the development of ectopic mineralization in patients with GACI [31], [32], [33], [34]. These case studies have yielded somewhat conflicting results; while improvement has been reported in some cases, in several studies the effects are not clear, and significant side effects from bisphosphonates have also been reported. The reasons for these diverse outcomes are not entirely clear but may relate to the fact that GACI is a complex clinical disorder with unpredictable progression, and there is no biomarker to measure the disease activity. The degree of disease progression is primarily assessed by occasional imaging studies of ectopic mineralization and the ultimate outcome of patient survival. Thus, the *asj-2J* mouse, genetically uniform on homogeneous strain background and under controlled environmental conditions, can serve as a platform to test pharmacological compounds with anti-mineralization properties, including various bisphosphonates.

In conclusion, the *asj-2J* mouse serves as a novel model to study molecular alterations in GACI, and it provides a platform to test various pharmacologic approaches for treatment of GACI and related, currently intractable, ectopic mineralization disorders.

## References

[1] Nitschke Y, Rutsch F (2012) Generalized arterial calcification of infancy and pseudoxanthoma elasticum: two sides of the same coin. Front Genet 3:302. 23269929 10.3389/fgene.2012.00302 PMC3529400

[2] Rutsch F, Nitschke Y, Terkeltaub R (2011) Genetics in arterial calcification: pieces of a puzzle and cogs in a wheel. Circ Res 109:578–592. 21852556 10.1161/CIRCRESAHA.111.247965 PMC3248761

[3] Rutsch F, Ruf N, Vaingankar S, Toliat MR, Suk A, et al (2003) Mutations in *ENPP1* are associated with ‘idiopathic’ infantile arterial calcification. Nature Genet 34:379–381. 12881724 10.1038/ng1221

[4] O’Neill WC, Sigrist MK, McIntyre CW (2010) Plasma pyrophosphate and vascular calcification in chronic kidney disease. Nephrol Dial Transplant 25:187–191. 19633093 10.1093/ndt/gfp362 PMC4326300

[5] Li Q, Uitto J (2013) Mineralization/anti-mineralization networks in the skin and vascular connective tissues. Am J Pathol 183:10–18. 23665350 10.1016/j.ajpath.2013.03.002 PMC3702739

[6] Mackenzie NC, Huesa C, Rutsch F, MacRae VE (2012) New insights into NPP1 function: lessons from clinical and animal studies. Bone 51:961–968. 22842219 10.1016/j.bone.2012.07.014

[7] Li Q, Guo H, Chou DW, Berndt A, Sundberg JP, et al (2013) Mutant *Enpp1^asj^* mouse as a model for generalized arterial calcification of infancy. Dis Model Mech 6:1227–1235. 23798568 10.1242/dmm.012765 PMC3759342

[8] Klement JF, Matsuzaki Y, Jiang QJ, Terlizzi J, Choi HY, et al (2005) Targeted ablation of the *Abcc6* gene results in ectopic mineralization of connective tissues. Mol Cell Biol 25:8299–8310. 16135817 10.1128/MCB.25.18.8299-8310.2005 PMC1234326

[9] Jiang Q, Uitto J (2012) Restricting dietary magnesium accelerates ectopic connective tissue mineralization in a mouse model of pseudoxanthoma elasticum *(Abcc6^−/−^)* . Exp Dermatol 21:694–699. 22897576 10.1111/j.1600-0625.2012.01553.x PMC3422765

[10] Li Q, Uitto J (2010) The mineralization phenotype in *Abcc6^−/−^* mice is affected by *Ggcx* gene deficiency and genetic background–a model for pseudoxanthoma elasticum. J Mol Med (Berl) 88:173–181. 19784827 10.1007/s00109-009-0522-8 PMC2879630

[11] Silva KA, Sundberg JP (2012) Necropsy methods. In: Hedrich HJeditor. The Laboratory Mouse. 2nd ed. London: Academic Press. pp. 779–806.

[12] Le Corre Y, Le Saux O, Froeliger F, Libouban H, Kauffenstein G, et al (2012) Quantification of the calcification phenotype of *Abcc6*-deficient mice with microcomputed tomography. Am J Pathol 180:2208–2213. 22469843 10.1016/j.ajpath.2012.02.007 PMC5691325

[13] Berndt A, Li Q, Potter CS, Liang Y, Silva KA, et al (2013) A single-nucleotide polymorphism in the Abcc6 gene associates with connective tissue mineralization in mice similar to targeted models for pseudoxanthoma elasticum. J Invest Dermatol 133:833–836. 23014343 10.1038/jid.2012.340 PMC4037127

[14] Tolouian R, Connery SM, O’Neill WC, Gupta A (2012) Using a filtration technique to isolate platelet free plasma for assaying pyrophosphate. Clin Lab 58:1129–1134. 23289181 PMC4733525

[15] Li Q, Price TP, Sundberg JP, Uitto J (2014) Juxta-articular joint-capsule mineralization in CD73 deficient mice: Similarities to patients with *NT5E* mutations. Cell cycle 13:2609–2615. 25486201 10.4161/15384101.2014.943567 PMC4614381

[16] Li Q, Chou DW, Price TP, Sundberg JP, Uitto J (2014) Genetic modulation of nephrocalcinosis in mouse models of ectopic mineralization: the Abcc6(tm1Jfk) and Enpp1(asj) mutant mice. Lab Invest 94:623–632. 24732453 10.1038/labinvest.2014.52 PMC4039617

[17] Nitschke Y, Rutsch F (2012) Genetics in arterial calcification: lessons learned from rare diseases. Trends Cardiovasc Med 22:145–149. 23122642 10.1016/j.tcm.2012.07.011

[18] Markello TC, Pak LK, St Hilaire C, Dorward H, Ziegler SG, et al (2011) Vascular pathology of medial arterial calcifications in NT5E deficiency: Implications for the role of adenosine in pseudoxanthoma elasticum. Mol Genet Metab 103:44–50. 21371928 10.1016/j.ymgme.2011.01.018 PMC3081917

[19] Zaka R, Williams CJ (2006) Role of the progressive ankylosis gene in cartilage mineralization. Curr Opin Rheumatol 18:181–186. 16462526 10.1097/01.bor.0000209432.36355.6e

[20] Bottger P, Hede SE, Grunnet M, Hoyer B, Klaerke DA, et al (2006) Characterization of transport mechanisms and determinants critical for Na+-dependent Pi symport of the PiT family paralogs human PiT1 and PiT2. Am J Physiol Cell Physiol 291:C1377–1387. 16790504 10.1152/ajpcell.00015.2006

[21] Miyamoto K, Haito-Sugino S, Kuwahara S, Ohi A, Nomura K, et al (2011) Sodium-dependent phosphate cotransporters: lessons from gene knockout and mutation studies. J Pharm Sci 100:3719–3730. 21567407 10.1002/jps.22614

[22] Jansen RS, Duijst S, Mahakena S, Sommer D, Szeri F, et al (2014) ABCC6-mediated ATP secretion by the liver Is the main source of the mineralization inhibitor inorganic pyrophosphate in the systemic circulation-brief report. Arterioscler Thromb Vasc Biol 34:1985–1989. 24969777 10.1161/ATVBAHA.114.304017 PMC6743317

[23] Li Q, Jiang Q, Uitto J (2008) Pseudoxanthoma elasticum: oxidative stress and antioxidant diet in a mouse model (Abcc6^−/−^). J Invest Dermatol 128:1160–1164. 18049453 10.1038/sj.jid.5701145 PMC3357057

[24] St Hilaire C, Ziegler SG, Markello TC, Brusco A, Groden C, et al (2011) *NT5E* mutations and arterial calcifications. N Engl J Med 364:432–442. 21288095 10.1056/NEJMoa0912923 PMC3049958

[25] Lorenz-Depiereux B, Schnabel D, Tiosano D, Hausler G, Strom TM (2010) Loss-of-function ENPP1 mutations cause both generalized arterial calcification of infancy and autosomal-recessive hypophosphatemic rickets. Am J Hum Genet 86:267–272. 20137773 10.1016/j.ajhg.2010.01.006 PMC2820166

[26] Okawa A, Nakamura I, Goto S, Moriya H, Nakamura Y, et al (1998) Mutation in *Npps* in a mouse model of ossification of the posterior longitudinal ligament of the spine. Nature Genet 19:271–273. 9662402 10.1038/956

[27] Takabayashi S, Seto S, Katoh H (2014) A new *Enpp1* allele, *Enpp1(ttw-Ham)*, identified in an ICR closed colony. Exp Anim 63:193–204. 24770645 10.1538/expanim.63.193 PMC4160980

[28] Sali A, Favaloro JM, Terkeltaub R, Goding JW (1999) Germline deletion of the nucleoside triphosphate pyrophosphohydrolase (NTPPPH) plasma cell membrane glycoprotein-1 (PC-1) produces abnormal calcification of periarticular tissues. In: Vanduffel L LReditor. Ecto-ATPases and related ectoenzymes. Maastricht, The Netherlands: Shaker Publishing. pp. 267–282.

[29] Babij P, Roudier M, Graves T, Han CY, Chhoa M, et al (2009) New variants in the *Enpp1* and *Ptpn6* genes cause low BMD, crystal-related arthropathy, and vascular calcification. J Bone Min Res 24:1552–1564. 10.1359/jbmr.090417 19419305

[30] Apschner A, Huitema LF, Ponsioen B, Peterson-Maduro J, Schulte-Merker S (2014) Zebrafish enpp1 mutants exhibit pathological mineralization, mimicking features of generalized arterial calcification of infancy (GACI) and pseudoxanthoma elasticum (PXE). Dis Model Mech 7:811–822. 24906371 10.1242/dmm.015693 PMC4073271

[31] Ramjan KA, Roscioli T, Rutsch F, Sillence D, Munns CF (2009) Generalized arterial calcification of infancy: treatment with bisphosphonates. Nat Clin Pract Endocrinol Metab 5:167–172. 19229237 10.1038/ncpendmet1067

[32] Rutsch F, Boyer P, Nitschke Y, Ruf N, Lorenz-Depierieux B, et al (2008) Hypophosphatemia, hyperphosphaturia, and bisphosphonate treatment are associated with survival beyond infancy in generalized arterial calcification of infancy. Circ Cardiovasc Genet 1:133–140. 20016754 10.1161/CIRCGENETICS.108.797704 PMC2794045

[33] Edouard T, Chabot G, Miro J, Buhas DC, Nitschke Y, et al (2011) Efficacy and safety of 2-year etidronate treatment in a child with generalized arterial calcification of infancy. Eur J Pediatr 170:1585–1590. 21932012 10.1007/s00431-011-1572-9

[34] Otero JE, Gottesman GS, McAlister WH, Mumm S, Madson KL, et al (2013) Severe skeletal toxicity from protracted etidronate therapy for generalized arterial calcification of infancy. J Bone Miner Res 28:419–430. 22972716 10.1002/jbmr.1752

